# Translation, cross-cultural adaptation, and validation of the Lung Transplant Quality of Life (LT-QOL) questionnaire for use in adult patients after lung transplantation into Portuguese Brazilian

**DOI:** 10.31744/einstein_journal/2026AO1882

**Published:** 2026-04-22

**Authors:** Felipe Farah Pinheiro Rodrigues, Soraia Micaela Silva, Grasiani Breggue Pires, Thaíse de Lucca Cappeline Dellabarba, Ricardo Kenji Nawa, Anya Sriram, Jonathan P. Singer, Luciana Maria Malosá Sampaio

**Affiliations:** 1 Postgraduate Program in Rehabilitation Sciences Universidade Nove de Julho São Paulo SP Brazil Postgraduate Program in Rehabilitation Sciences, Universidade Nove de Julho, São Paulo, SP, Brazil.; 2 Hospital Israelita Albert Einstein São Paulo SP Brazil Hospital Israelita Albert Einstein, São Paulo, SP, Brazil.; 3 Department of Medicine University of California San Francisco California United States Department of Medicine, University of California, San Francisco, California, United States.

**Keywords:** Quality of life, Quality indicators, health care, Lung transplantation, Surveys and questionnaires, Translations

## Abstract

**Objective:**

To translate, cross-culturally adapt, and validate the Lung Transplant Quality of Life questionnaire into Brazilian Portuguese.

**Methods:**

This observational study enrolled 50 participants. Translation and cross-cultural adaptation were conducted in accordance with established guidelines for self-report measures and the Consensus-based Standards for the Selection of Health Measurement Instruments (COSMIN). The measurement properties assessed included internal consistency, agreement, intra-rater reliability, and convergent validity. Convergent validity was evaluated by comparing the Lung Transplant Quality of Life with the Short Form Health Survey (SF-36) and the Saint George’s Respiratory Questionnaire.

**Results:**

The Lung Transplant Quality of Life showed adequate internal consistency (Cronbach’s alpha=0.86) and excellent intra-rater reliability (intraclass correlation coefficient=0.93). Agreement analysis yielded a standard error of measurement of 8.72. Bland-Altman analysis revealed low mean differences (0.8) between Lung Transplant Quality of Life scores and acceptable limits of agreement (95%CI=-16.9–15.3). Spearman’s correlation showed excellent convergent validity with the SF-36 and good validity with the Saint George’s Respiratory Questionnaire in both test and retest phases.

**Conclusion:**

The Brazilian Portuguese version of the Lung Transplant Quality of Life is a valid and reliable instrument for assessing health-related quality of life in lung transplant patients. Further research is warranted to develop a shorter version while preserving its measurement properties.

## HIGHLIGHTS

■ First Brazilian Portuguese version of the LT-QOL questionnaire.

■ Translation and cross-cultural adaptation followed COSMIN recommendations.

■ Strong internal consistency (Cronbach’s alpha=0.86).

■ Excellent intra-rater reliability (ICC=0.93).

■ High convergent validity with SF-36 and good correlation with SGRQ.

■ Provides a disease-specific tool for assessing quality of life after lung transplantation in Brazil.

■ Supports future clinical monitoring and research on transplant outcomes.

## In Brief

Lung transplantation significantly affects patients’ quality of life, yet Brazil previously lacked a validated, disease-specific instrument to assess this outcome. In this study, we translated, culturally adapted, and validated the LT-QOL questionnaire for Brazilian Portuguese. The instrument demonstrated strong reliability, good agreement, and excellent convergent validity compared with the SF-36 and SGRQ. The Brazilian LT-QOL provides clinicians and researchers with a robust and practical tool to evaluate healthrelated quality of life in adult lung transplant recipients in Brazil.

## Graphical abstract

**Figure:**
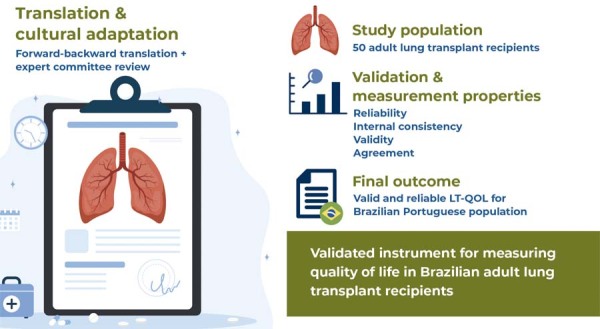

## INTRODUCTION

The outcome of lung transplantation has traditionally been analyzed based on graft function and patient survival. However, the assessment of quality of life has increasingly been recognized as an essential outcome measure for guiding postsurgical interventions.^(1,2)^ Objective measurement and quantification of quality of life and survival after lung transplantation are critical for analyzing the effectiveness of therapeutic interventions and justifying the high costs associated with this complex surgical procedure.^(3-6)^

The absence of a disease-specific instrument to assess the quality of life in post-lung transplant patients, particularly one that adequately represents patient-reported symptoms, highlights the need for a questionnaire that is translated, cross-culturally adapted, and validated for the Portuguese/Brazilian language for application in this population, with the possibility of providing better understanding and assistance in evaluating the efficacy of the therapies used.

Important domains for lung transplant recipients, such as depression and anxiety, extrapulmonary and immunosuppressive-related symptoms, and neurocognitive deficits, are insufficiently addressed by currently available generic instruments or tools developed for respiratory diseases.^7^

## OBJECTIVE

The objective of the present study was to translate, cross-culturally adapt, and validate the “Lung transplant Quality of Life” (LT-QOL) questionnaire into the Portuguese-Brazilian language for use in adult patients after lung transplantation. Additionally, the study aimed to test the final translated version of the “LT-QOL” instrument by evaluating intra-rater reliability and agreement in the outpatient setting with a 14-day interval between assessments, as well as to assess internal consistency and convergent validity of the translated version.

## METHODS

This single-center prospective study was conducted at the *Hospital Israelita Albert Einstein* (HIAE), São Paulo, SP, Brazil. Participants were recruited from the post-lung transplant rehabilitation outpatient clinic at the HIAE Vila Mariana Unit. All eligible participants were approached by a trained interviewer between February 2023 and November 2023 and were provided with comprehensive information necessary to understand the study. The study protocol was approved by the Human Research Ethics Committee (CEP) of *Universidade Nove de Julho* (CAAE: 67592623.6.0000.5511; # 5.983.617), the proposing institution, and by the CEP of the *Hospital Israelita Albert Einstein* (CAAE: 67592623.6.3001.0071; # 6.065.050), a co-participating institution. All participants were informed about the study purpose, procedures, and potential risks and subsequently provided written informed consent prior to participation.

### Participants

Individuals who met the following criteria were invited to participate in the study: adults over 18 years of age who had undergone lung transplant surgical treatment (unilateral or bilateral) and who were currently enrolled in or had previously completed the post-lung transplant rehabilitation program at the HIAE rehabilitation outpatient clinic. Participants actively engaged in the rehabilitation program were recruited during physiotherapy sessions at the outpatient clinic, whereas patients who had completed the rehabilitation program were recruited through telephone contact. Individuals who demonstrated deficits in understanding the questionnaires or who refused to respond to any of the questionnaires used in this study were excluded. All questionnaires were administered verbally by a single interviewer, who held a bachelor’s degree in physical therapy and had 19 years of clinical experience.

### Study design

This study analyzed the measurement properties and cross-cultural adaptation of the instrument. To evaluate concurrent validity, we also administered the Medical Outcomes Study Short Form (SF-36) and the Saint George’s Respiratory Questionnaire (SGRQ). The SF-36 consists of 36 items distributed across eight domains (functional capacity, physical aspects, pain, general health status, vitality, social aspects, emotional aspects, and mental health), with scores ranging from 0 to 100, where zero represents the worst general health status, and 100 represents the best health status.^(9)^ The SGRQ consists of 50 items divided into three domains (symptoms, activities, and impact), which assess social and psychological functioning that may affect the patient’s lifestyle.^(10)^ Each domain has a maximum possible score, and the points of each response are summed and expressed as a percentage of this maximum.

The SF-36 and SGRQ were selected because they are well-known and frequently used questionnaires for assessing the quality of life of patients with respiratory diseases, and because translated and validated Portuguese-Brazilian versions are available, in addition to the “LT-QOL.” The authors of the latter authorized its translation into Brazilian Portuguese. The translation methodology followed the *“Guidelines for the Process of Cross-cultural Adaptation of Self-Report Measures”.*^(11)^

### LT-QOL Score

The LT-QOL comprises four domains: symptoms (pulmonary, gastrointestinal, and neuromuscular); health perception (questions related to treatment burden and concerns about future health); functioning (questions addressing cognitive limitations and sexual health); and well-being (questions related to anxiety, depression, health problems, and overall quality of life). Lower scores indicate better health, except for the domain assessing “Overall quality of health,” where higher scores denote better health. The LT-QOL consists of 60 items distributed across the domains described above, with each item using a scale ranging from 1 to 5. For all domains except one, higher scores (closer to 5) indicate poorer health, reflecting more symptoms, greater suffering, or increased limitations. The exception is the item assessing “overall quality of life,” where a higher score represents better quality of life.

No cutoff score is defined for interpreting better or worse quality of life; analysis is performed using the mean score for each domain. Evaluations should be conducted by domain to ensure accurate interpretation of the results.

### Translation and adaptation

The translation process followed six phases: “Phase I” – initial translation; “Phase II” – synthesis of translations; “Phase III” – reverse translation; “Phase IV” – expert committee review; “Phase V” – pre-testing of the final version; and “Phase VI” – submission and consideration of all written reports.^(11)^

#### Phase I – Initial Translation

Phase I involved translating the questionnaire from its original English version into Portuguese-Brazilian. Two translators (“TR1” and “TR2”) independently translated the questionnaire without any communication or contact between them.

None of the translators had previous contact with the questionnaire to be translated, ensuring that they had no prior knowledge of the content. One translator was a native English speaker fluent in Portuguese-Brazilian, and the other was a native Portuguese-Brazilian speaker fluent in English. “TR1” had previous knowledge of health-related concepts and terminology, enabling a more technically accurate translation. In contrast, “TR2” had only lay knowledge of health-related topics and no familiarity with technical terms.

The two versions translated into Portuguese-Brazilian (T1 and T2) included comments from the translators to document uncertainties and possible discrepancies, which were then discussed in “Phase II.”

#### Phase II – Synthesis of translations

Phase II involved synthesizing the translations (“T1” and “T2”) produced in Phase I. Under the coordination of the principal investigator, the translators met to produce a unified version (“T1-2”), discussing and resolving any discrepancies to reach consensus. The synthesis process was fully documented in a report, including all justifications for adjustments made during this stage.

#### Phase III – Reverse Translation

In Phase III, a third translator (“TR3”), a native English speaker with no prior knowledge of the original questionnaire, performed a back translation of the unified Portuguese-Brazilian version (“T1-2”) into English (“RT1-2”). The back-translated version (“RT1-2”) was sent to the original author and study team responsible for the questionnaire for proper validation of the content. The original author and study team identified specific items in which the content of the back translation did not fully reflect the intended meaning of the original items. Based on this feedback, modifications were made to the content of the “RT1-2” version, and “Phase II” was resumed to allow discussion and revision of the identified points.

#### Phase IV – Expert Committee

In Phase IV, the reverse-translated version (“RT1-2”) was reviewed by a committee of experts to assess the cross-cultural equivalence of the questionnaire. The committee was composed of four professionals: two health professionals, one language professional, one methodologist, and the translators involved in “Phases I and II” (“TR1,” “TR2,” and “TR3”). The authors of the original questionnaire maintained contact with the committee throughout this process.

With access to the original questionnaire, all translated versions, and their corresponding reports and observations, the objective of this committee was to review the translations, resolve any discrepancies, and develop a pre-final version for field testing. This committee aimed to achieve equivalence between the original language and the translated versions across the following areas:

Semantic equivalence – assessed grammatical structure and vocabulary to determine whether words retained the same meaning after translation; Idiomatic equivalence – addressed difficulties related to translating colloquial expressions from the English language; Experiential or cultural equivalence – sought to align the terms used with the lived experiences of the target population; and Conceptual equivalence – ensured that the underlying concepts of the terms were appropriately adapted between both languages.

#### Phase V – Pre-testing of the final version

In this phase, the “RT1-2” version of the questionnaire was administered to a population of 30 individuals. After completing the questionnaire, each participant was interviewed to assess their level of understanding of each item and their selected responses. A scale ranging from “0” to “5” points was applied, in which “0” indicated no comprehension and “5” indicated complete comprehension. The meanings of the items and answers were explored to confirm that the adapted version maintained equivalence with the original version. The distribution of responses was also examined to identify a high proportion of missing items or patterns of single responses.

#### Phase VI – Submission and appraisal

In Phase VI, a comprehensive audit of the entire process was conducted, during which all reports and translated versions were presented. The original questionnaire study team reviewed all phases to confirm that the procedures had been properly followed and were in accordance with the recommended cross-cultural adaptation process.

## Measurement properties

Evaluations related to measurement properties or outcomes of an instrument were performed. For statistical analysis, the SPSS software for Windows (version 22; SPSS Inc.; USA, Armonk, NY) was used. The Shapiro-Wilk test was applied to assess the normality of the data. Parametric variables were described as mean and standard deviation (SD). Categorical variables were described as absolute numbers, percentages, and frequencies. Clinimetric analyses included assessment of internal consistency, agreement, intra-rater reliability, convergent validity, and ceiling-floor effects.

## Sample size

For the sample size calculation, we followed the recommendations of *the “COnsensus-based Standards for the selection of health Measurement INstruments*” (COSMIN) guideline, which suggests that studies with the objective of performing an adequate evaluation of internal consistency, agreement, reliability and construct validity can be classified as having an “adequate” sample when composed of 100 or more participants; a “good” sample when composed of 50 to 99 participants; a “moderate” sample when composed of 30 to 49 participants; and a “small” sample when composed of fewer than 30 participants.^(12)^

## RESULTS

A total of 61 candidates were assessed for eligibility in this study. Of these, 50 met the inclusion criteria, 10 refused to complete the questionnaires used in the study, and one candidate provided incomplete questionnaire responses. Thus, 50 candidates were included in the final analysis.

The mean age of the participants was 50.20±15.07 years, consisting of 56% females and 44% males. The mean body mass index of the participants was 23.58kg/m^2^±3.67kg/m^2^. Regarding self-reported race, 68% identified as White, non-Hispanic, 28% as White, Hispanic, 2% as Asian, and 2% as Black (Table 1).

**Table 1:** Demographic and clinical characteristics of the sample

Variables	Mean (SD), n (%)
Anthropometric	
Age (years)	50.20 (15.07)
BMI (kg/m^2^)	23.58 (3.67)
Gender, n (%)	
Female	28 (56)
Male	22 (44)
Race, n (%)	
White, non-Hispanic	34 (68)
Asian	1 (2)
White, Hispanic	14 (28)
Black	1 (2)
Destination Hospital discharge, n (%)	
Residence	34 (68)
Transfer to another hospital	16 (32)
Type Tx, n (%)	
Unilateral	6 (12)
Bilateral	44 (88)
Diagnosis, n (%)	
COPD	13 (26.0)
Idiopathic pulmonary fibrosis	8 (16)
Cystic fibrosis	6 (12.0)
Pulmonary arterial hypertension	5 (10.0)
Bronchiectasis	3(6.0)
Sarcoidosis	3 (6.0)
Bronchiolitis	3(6.0)
Other	3 (6.0)
Hypersensitivity pneumonia	2(4.0)
Post-covid-19	2 (4.0)
Primary Ciliary Dyskinesia	1 (2.0)
Systemic Lupus Erythematosus	1(2.0)
Organ waiting time (in days)	588±531.37

SD: standard deviation; BMI: body mass index; Tx: transplantation; COPD: chronic obstructive pulmonary disease.

Regarding the type of transplant performed, 88% of the sample (44 participants) underwent bilateral lung transplantation, whereas 12% (6 participants) underwent unilateral lung transplantation.

Chronic obstructive pulmonary disease was the most frequent primary indication for lung transplantation, accounting for 26% of cases, followed by idiopathic pulmonary fibrosis (16%), cystic fibrosis (12%), and pulmonary arterial hypertension (10%).

Study participants performed the 6-minute walk test (6MWT) at the time of listing for lung transplantation (pre-transplant phase) and after completion of 36 outpatient physical therapy sessions following surgical treatment (post-transplant phase). These data are presented in table 2, along with the domain-specific scores for each questionnaire administered and readministered during the study (first and second applications, with a 14-day interval). Regarding the 6MWT, results are reported for 43 patients, as seven patients did not complete the 36-session rehabilitation program and were therefore classified as dropouts, as recommended by the rehabilitation service of *Hospital Israelita Albert Einstein*.

**Table 2:** Exercise capacity data (pre- and post-transplant) and LT-QOL, SF-36, and SGRQ questionnaires scores

Exercise capacity	Mean (SD)
6min WT	
Pre-transplant (meters)	313.43 (98.16)
Post-transplant (meters)	533.25 (88.22)
HRQoL	
SF-36 (Q1)	
Domain 1 (Functional capacity)	76.00 (28.27)
Domain 2 (Limitation due to physical aspects)	58.5 (46.46)
Domain 3 (Pain)	78.86 (24.88)
Domain 4 (Limitation by emotional aspect)	62.00 (46.19)
Domain 5 (Vitality)	70.3 (21.27)
Domain 6 (Social aspects)	72.94 (30.47)
Domain 7 (Mental health)	75.2 (20.24)
Domain 8 (General health status)	73.62 (18.79)
SF-36 (Q2)	
Domain 1 (Functional capacity)	75.6 (27.36)
Domain 2 (Limitation due to physical aspects)	64.00 (45.22)
Domain 3 (Pain)	75.56 (25.23)
Domain 4 (Limitation by emotional aspect)	66.68 (44.68)
Domain 5 (Vitality)	71.40 (20.75)
Domain 6 (Social aspects)	73.66 (31.02)
Domain 7 (Mental health)	76.88 (19.04)
Domain 8 (General health status)	70.30 (18.39)
SGRQ (Q1)	
Domain 1 (Symptoms)	9.50 (18.25)
Domain 2 (Activity)	17.30 (28.05)
Domain 3 (Impacts)	13.40 (19.38)
**Exercise capacity**	**Mean (SD)**
SGRQ (Q2)	
Domain 1 (Symptoms)	9.20 (18.70)
Domain 2 (Activity)	20.9 (28.29)
Domain 3 (Impacts)	15.35 (18.72)
LT- QOL (Q1) symptoms	
Pulmonary symptoms	1.66±0.59
Shortness of breath	1.74±0.79
Cough	1.66±0.66
Gastrointestinal symptoms	1.51±0.52
Eating / Aspiration problem	1.28±0.65
Lack of interest in eating	1.65±0.79
Upper GI symptoms	1.54±0.60
Lower GI symptoms	1.68±0.93
Neuromuscular symptoms	2.06±0.96
Health perceptions	
Treatment burden	1.41±0.65
Worry about future health	2.74±1.12
Functioning	
Cognitive limitations	2.17±0.98
Sexual problems	2.00±1.15
Well being	
Anxiety / depression	2.09±0.99
Anxiety	1.81±0.79
Depression	1.85±0.99
Health distress	2.55±1.57
General quality of life	3.94±0.89
LT- QOL(Q2) Symptoms	
Pulmonary symptoms	1.61±0.57
Shortness of breath	1.69±0.77
Cough	1.58±0.64
Gastrointestinal symptoms	1.55±0.56
Eating / Aspiration problem	1.30±0.72
Lack of interest in eating	1.68±0.84
Upper GI symptoms	1.66±0.75
Lower GI symptoms	1.60±0.83
Neuromuscular symptoms	2.12±0.89
Health perceptions	
Treatment burden	1.38±0.68
Worry about future health	2.54±1.05
Functioning	
Cognitive limitations	2.09±1.03
Sexual problems	1.85±1.03
Well being	
Anxiety / depression	1.98±0.96
Anxiety	1.75±0.73
Depression	1.84±0.92
Health distress	2.4±1.65
General quality of life	3.88±0.98

SD: standard deviation; 6MWT: 6-minute walk test; HRQoL: health-related quality of life; LT-QOL: Lung transplant quality of life; SF-36: Medical Outcome study 36–Item Short-Form Health Survey; SGRQ: Saint George respiratory questionnaire; Q1: first administration of the questionnaire; Q2: second administration of the questionnaire (time interval between Q1 and Q2 is 14 days).

### Internal consistency and reliability

Test-retest analysis of the LT-QOL demonstrated adequate reliability and internal consistency. Cronbach’s alpha was 0.86, indicating good internal consistency.^(13)^ Reliability was assessed using the intraclass correlation coefficient (ICC), which reached 0.93 and was classified as excellent.^(14)^ These results, summarized in table 3, were obtained for each domain of the questionnaire.

**Table 3:** LT-QOL Reproducibility analysis

	ICC			α-		
	Test	Retest	(95%CI)	Cronbach’s	SEM^‡^	MMD^§^
Symptoms						
Pulmonary symptoms	1.66±0.59	1.61±0.57	0.89 (0.82–0.94)	0.89	0.20	0.54
Shortness of breath	1.74±0.79	1.69±0.77	0.91 (0.85–0.95)	0.95	0.24	0.66
Cough	1.66±0.66	1.58±0.64	0.76 (0.62–0.86)	0.86	0.32	0.90
Gastrointestinal symptoms	1.51±0.52	1.55±0.56	0.81 (0.68–0.88)	0.89	0.23	0.63
Eating / Aspiration problem	1.28±0.65	1.30±0.72	0.92 (0.86–0.95)	0.95	0.18	0.51
Lack of interest in eating	1.65±0.79	1.68±0.84	0.81 (0.68–0.88)	0.89	0.34	0.95
Upper GI symptoms	1.54±0.60	1.66±0.75	0.58 (0.36–0.73)	0.73	0.39	1.08
Lower GI symptoms	1.68±0.93	1.60±0.83	0.87 (0.78–0.92)	0.93	0.34	0.93
Neuromuscular symptoms	2.06±0.96	2.12±0.89	0.80 (0.67–0.88)	0.88	0.43	1.19
Health perceptions						
Treatment burden	1.41±0.65	1.38±0.68	0.84 (0.74–0.91)	0.91	0.26	0.72
Worry about future health	2.74±1.12	2.54±1.05	0.81 (0.68–0.88)	0.89	0.49	1.35
Functioning						
Cognitive limitations	2.17±0.98	2.09±1.03	0.82 (0.71–0.89)	0.90	0.42	1.15
Sexual problems	2.00±1.15	1.85±1.03	0.74 (0.58–0.84)	0.85	0.59	1.63
Well being						
Anxiety / depression	2.09±0.99	1.98±0.96	0.77 (0.64–0.86)	0.87	0.47	1.32
Anxiety	1.81±0.79	1.75±0.73	0.85 (0.75–0.91)	0.92	0.31	0.85
Depression	1.85±0.99	1.84±0.92	0.80 (0.67–0.88)	0.88	0.44	1.23
Health distress	2.55±1.57	2.40±1.65	0.92 (0.86–0.95)	0.95	0.44	1.23
General quality of life	3.94±0.89	3.88±0.98	0.34 (0.07–0.56)	0.51	0.72	2.00

^‡^ Standard Error of Measurement (SEM) is a statistical measure that quantifies the accuracy of a sample mean relative to the population mean. ^§^ Minimal Meaningful Difference (MMD) represents the smallest change in a measurement that can be interpreted as a true change.

In LT-QOL, lower scores indicate better health, except for the domain “Overall quality of health,” where higher scores denote better health.

### Agreement

The standard error of measurement (SEM) reflects the error associated with the instrument and is calculated by multiplying the standard deviation by the square root of 1 minus the intraclass correlation coefficient [ SD ×√(1−CCI)] . The Minimal Meaningful Difference (MMD), defined as the smallest change in measurement that can be interpreted as a true change, was calculated using the formula $\mathrm{MMD}=1.96\times \sqrt{2}\times \mathrm{SEM}$ .^(15)^ The MMD was also expressed as a percentage (“DCM%”), which provides an independent estimate of the measurement units and represents the relative magnitude of random measurement error. For this purpose, the minimum detectable percentage change was calculated as: (minimum detectable change/observed mean of the total score of the analyzed test) × 100. A minimum detectable percentage change of <30% was considered acceptable, whereas values <10% were considered excellent.

Descriptive statistics for the second-order lung transplant scales are presented in table 3. Cronbach’s alpha for the LT-QOL scales ranged from 0.51 to 0.95, with all but two scales achieving values ≥0.80, indicating excellent internal consistency. The scale scores ranged from 1 to 5. Mean scores (±SD) on scales for which higher scores represent worse health ranged from 1.30 (±0.72) on the Eating/Aspiration problem scale to 2.74 (±1.12) on the Worry About Future Health scale. Only five of these scales had mean values greater than 2.0. For the general quality of life scale, where higher scores indicate better health, the mean score was 3.94 (±0.89). Overall, participants’ HRQL across the second-order scales was generally high.

Ceiling and floor effects were considered absent because, in the frequency analysis, less than 15% of the participants achieved the maximum or minimum possible score in any scale. All results and classifications from the clinimetric analyses are described in table 4 for better visualization. The mean time to complete the questionnaires was 20 minutes for the LT-QOL, 16 minutes for the SGRQ, and 10 minutes for the SF-36.

**Table 4:** Classification of the clinimetric analyses of the LT-QOL questionnaire

Properties	Values	Classification
Internal consistency Cronbach’s Alpha	0.86	Adequate
Measurement error Standard Error of Measurement Minimum Difference Detected	Values 8.72 8.19	Good Adequate
Reproducibility CCI, (95%CI)	0.93 (0.88–0.96)	Excellent
Ceiling and floor effect	Absence	Absence

ICC: Intraclass Correlation Coefficient; 95% CI: 95% confidence interval; r: Spearman’s correlation, p≤0.001; Q1: first administration of the questionnaire; Q2: second administration of the questionnaire.

## DISCUSSION

The need for a specific instrument to assess the quality of life of adult patients after lung transplantation in the Portuguese-Brazilian version represents an important tool for achieving a better interpretation of lung transplant outcomes.

Despite the existence of different instruments for assessing health-related quality of life, no validated tool is available for use in lung transplant recipients in Brazil. As a result, different centers specializing in lung transplantation use generic tools such as the SF-36 and SGRQ, which are also applied to assess the health-related quality of life (HRQoL) of patients with various respiratory diseases.^(16,17)^

The use of a single interviewer throughout all stages of questionnaire administration may be considered a limitation of this study, as this approach has the potential to introduce interviewer-related bias or influence participants’ responses, particularly for more sensitive items. However, this approach also offers an important methodological advantage: the standardization of administration procedures. By using the same interviewer, variability related to differences in communication style, explanation of items, and interaction with participants was minimized, thereby enhancing the consistency and internal reliability of data collection.

The results reinforce the importance of the LT-QOL for assessing the quality of life of lung transplant recipients, as this questionnaire includes essential domains that are not captured by more general instruments such as the SF-36. These domains include concerns about the future, transplant-specific symptoms, and neuropsychological aspects such as depression, anxiety, and cognitive impairment, making the LT-QOL a more comprehensive and sensitive tool for addressing the specific characteristics of this population.^(18)^Thus, the LT-QOL was developed using 10 scales designed to evaluate symptoms, perceptions of health, functionality status, and well-being.

With four domains: symptoms (pulmonary, gastrointestinal, and neuromuscular); perceived health (issues related to treatment burden and concerns about future health); functionality (issues related to cognitive limitations and sexual health); and well-being (issues related to anxiety, depression, health problems, and overall quality of life), the LT-QOL demonstrates a better quality of life in individuals with lower scores (except for the “Overall Quality of Life” domain, in which higher scores denote better health status). Therefore, individuals reporting a better quality of life present lower scores than those reporting a poorer quality of life.

Internal consistency assessed through test-retest analysis was adequate for the questionnaire, with a Cronbach’s α of 0.86. This measurement property is particularly relevant for questionnaires designed to evaluate a concept using multiple items/domains.^(13)^In the original study, the authors aimed to achieve a Cronbach’s α greater than 0.07 for each LT-QOL scale and reported values ranging from 0.75 to 0.95.^(18)^Based on the adequate internal consistency demonstrated in the present study, together with the values reported in the original validation study, we can infer that the LT-QOL questionnaire shows an appropriate relationship among its evaluation items.

The results also demonstrated high reproducibility. Reliability assessed using the ICC was 0.93 (95%CI= 0.88–0.96), which is considered excellent. Evaluations were performed for each domain in the questionnaire, and the results demonstrate that the instrument reliably reproduces symptom-related outcomes.

However, to ensure comprehensive coverage of the domains described above, the questionnaire consists of 60 questions, which requires a longer administration time compared with other commonly used questionnaires.

Given the absence of disease-specific questionnaires for assessing HRQoL in Brazilian adult patients undergoing lung transplantation, we used the SF-36 and SGRQ questionnaires to evaluate the concurrent validity of the LT-QOL questionnaire. These tools were selected because they are commonly used to assess the HRQoL in patients with respiratory diseases, in addition to being easily applicable in clinical and research settings.^(3,9,10,15,16)^

The concurrent validity of the LT-QOL questionnaire, assessed using Spearman’s correlation, was considered good at the first administration (Q1) when compared with the SF-36 (r=–0.773; p=0.000) and also good when compared with the SGRQ (r=0.585; p=0.000). In the second administration (Q2), the LT-QOL again demonstrated strong concurrent validity, showing correlations of r=–0.785 (p=0.000) with the SF-36 and r=0.632 (p=0.000) with the SGRQ. These results confirm that the translated and cross-culturally adapted LT-QOL questionnaire is a valid tool for assessing HRQoL in adult lung transplant recipients and underscore its practical value for clinical use in patient management.

Agreement of the instrument, assessed by the SEM (8.72), was considered “good,” with a minimum difference of 8.19. This result shows that the LT-QOL questionnaire has adequate sensitivity to detect changes in HRQoL among lung transplant recipients over different time intervals. The limit of agreement, analyzed using the Bland-Altman technique, showed a low mean difference (0.8) and acceptable limits (95%CI=-16.9–15.3), further supporting the good test–retest agreement observed.

No ceiling or floor effects were identified in our study, as fewer than 15% of participants achieved either the highest or lowest possible LT-QOL scores. This finding indicates that the questionnaire adequately discriminates among different levels of health-related quality of life within the study population. We believe that the relatively large number of items (60), combined with multiple response options for each item, likely contributed to the wide distribution of scores and reduced the likelihood of ceiling or floor effects.

Regarding the 6MWT, correlation with the questionnaire scores was not feasible because the test was performed at a different time point from the administration of the questionnaires used in the study. In this context, the 6MWT was used to characterize the sample.

Despite encompassing a larger number of questions (60 questions), the LT-QOL can be considered an important instrument for the comprehensive assessment of quality of life in adult patients after lung transplantation. The questionnaire includes questions specifically addressing each evaluated domain, allowing better representation of symptoms, perception of health, functionality, and well-being in this population, and consequently providing a more specific and reliable assessment. As such, the LT-QOL represents a valuable adjunct instrument for therapeutic decision-making.

In line with the original LT-QOL authors, we acknowledge that the length of the full instrument may limit its broader use across lung transplant centers, as administration requires more time compared with other HRQoL questionnaires. Although the development of a formal short-form version was beyond the scope of the present study – as previously clarified to the reviewer – we agree that strategies to reduce respondent burden warrant consideration in future research. As an intermediate and methodologically appropriate approach, future studies may consider administering only the subscales most relevant to their specific objectives. For example, studies primarily focused on respiratory outcomes may choose to apply the Pulmonary Symptoms subscale while excluding domains such as Cognitive Limitations when they are not central to the study aims.

The original LT-QOL instrument was developed by Singer et al.,^(18)^ who kindly authorized its use for this study. The full Brazilian Portuguese version, including the translated and cross-culturally adapted questionnaire, is provided in Table 1S , Supplementary Material.

## CONCLUSION

We conclude that the LT-QOL questionnaire was successfully translated and cross-culturally adapted for use in adult patients undergoing lung transplantation in Brazil, as supported by the clinimetric results presented in this study. It is noteworthy that the instrument contains more items than other quality of life questionnaires commonly used in this population, reflecting its broader and more detailed approach to assessing the quality of life of lung transplant recipients.

## SUPPLEMENTARY MATERIAL

SUPPLEMENTARY MATERIAL
