# Supplementary material for: Translation, cross-cultural adaptation, and validation of the Lung Transplant Quality of Life (LT-QOL) questionnaire for use in adult patients after lung transplantation into Portuguese Brazilian
Source: Einstein (Sao Paulo). 2026 Apr 22;24:eAO1882. doi: 10.31744/einstein_journal/2026AO1882 (PMC13128239; doi:10.31744/einstein_journal/2026AO1882)
Supplement: SUPPLEMENTARY MATERIAL [file 2317-6385-eins-24-eAO1882-suppl01.pdf]

SUPPLEMENTARY MATERIAL

Translation, cross-cultural adaptation, and validation of the Lung Transplant Quality of Life (LT-QOL) questionnaire for use in adult patients after lung transplantation into Portuguese Brazilian

Felipe Farah Pinheiro Rodrigues, Soraia Micaela Silva, Grasiani Breggue Pires, Thaíse de Lucca Cappeline Dellabarba, Ricardo Kenji Nawa, Anya Sriram, Jonathan P. Singer, Luciana Maria Malosá Sampaio

DOI: 10.31744/einstein_journal/2026AO1882

Table 1S. Lung Transplant Quality of Life (LT-QOL) questionnaire, translated and cross-culturally adapted

QUESTIONÁRIO DE QUALIDADE DE VIDA EM TRANSPLANTE PULMONAR (LT-QOL) TRADUZIDO E ADAPTADO TRANSCULTURALMENTE

Agradecemos imensamente sua participação em nosso estudo. Por favor, siga para iniciar a pesquisa.

Pensando nas últimas 4 semanas, quantas vezes você sentiu o seguinte quando NÃO tinha infecção ou rejeição?					
	**Nem um pouco**	**Apenas quando tinha uma infecção**	**Poucos dias no mês**	**Muitos dias no mês**	**Quase todos os dias**
1. Tinha falta de ar.	1	2	3	4	5
2. Senti aperto no peito.	1	2	3	4	5
3. Eu tossi.	1	2	3	4	5
4. Eu cuspi catarro.	1	2	3	4	5
5. Tive episódio de chiado.	1	2	3	4	5
	**Todo dia**	**Quase todo dia**	**3 ou 4 dias por semana**	**1 ou 2 dias por semana**	**Nenhum dia**
6. Nos últimos 3 meses, quantos dias bons (com poucos problemas pulmonares/respiratórios) você teve?	1	2	3	4	5
	**Nenhum episódio**	**1 episódio**	**2 episódios**	**3 episódios**	**Mais de 3 episódios**
7. Nos últimos 3 meses, quantos episódios severos ou muito ruins de problemas pulmonares/respiratórios você teve?	1	2	3	4	5
**Abaixo está uma lista de sintomas e condições que você pode ter experimentado. Nas últimas 4 semanas, quantas vezes você experimentou o seguinte?**	**Nunca**	**Uma ou duas vezes**	**Algumas vezes**	**Muitas vezes**	**Frequentemente**
8. Tive dificuldade em engolir comida.	1	2	3	4	5
9. Tive dificuldades em engolir líquidos.	1	2	3	4	5
10. Engasguei ao engolir.	1	2	3	4	5
11. Me incomodei pelo gosto da comida.	1	2	3	4	5
12. Tive pouco apetite.	1	2	3	4	5
13. Senti náusea.	1	2	3	4	5
14. Senti desconforto ou dor na área estomacal.	1	2	3	4	5
15. Tive inchaço ou cólicas na área estomacal.	1	2	3	4	5
16. Tive constipação.	1	2	3	4	5
17. Tive diarréia.	1	2	3	4	5
18. Tive medo de ficar longe do banheiro.	1	2	3	4	5
19. Tive tremores nas mãos.	1	2	3	4	5
20. Senti fraqueza nos músculos das pernas.	1	2	3	4	5
21. Tive dormência e formigamento nas mãos ou pés.	1	2	3	4	5
22. Senti desconforto nas mãos ou pés (dor, câimbras, queimação, etc.)	1	2	3	4	5

continue...

...Continuation

Estas perguntas são sobre seu regime de tratamento (medicamentos, visitas à clínica e exames como raio-X, broncoscopias) nas últimas 4 semanas.	Nenhum pouco	Muito pouco	Um pouco	Muito	Muitíssimo
23. Os efeitos do tratamento tem sido piores do que eu imaginei.	1	2	3	4	5
24. Até que ponto seus tratamentos (incluindo medicamentos) tornaram a sua vida diária mais difícil?	1	2	3	4	5
25. Quão difícil foi para você seguir o seu tratamento (incluindo medicamentos) todos os dias?	1	2	3	4	5
**Ao longo das últimas 4 semanas, o quanto cada declaração se aplica a você?**	**Nenhum pouco**	**Muito pouco**	**Um pouco**	**Muito**	**Muitíssimo**
26. Eu me preocupo que meu transplante de pulmão não funcione ou que eu tenha rejeição do transplante.	1	2	3	4	5
27. Eu me preocupo em pegar infecções.	1	2	3	4	5
28. Por causa do meu transplante pulmonar, tive dificuldade em planejar para o futuro.	1	2	3	4	5
29. Eu me preocupo que minha saúde piore.	1	2	3	4	5
30. Senti incerteza sobre a minha saúde futura.	1	2	3	4	5
**Ao longo das últimas 4 semanas, com que frequência você foi incomodado pelos seguintes problemas?**	**Nunca**	**Uma ou duas vezes**	**Poucas vezes**	**Muitas vezes**	**Frequentemente**
31. Sentindo nervoso, ansioso ou impaciente.	1	2	3	4	5
32. Não ser capaz de parar ou controlar a ansiedade.	1	2	3	4	5
33. Me preocupando demais com coisas diferentes.	1	2	3	4	5
34. Dificuldade em relaxar.	1	2	3	4	5
35. Estar tão inquieto que é difícil ficar parado.	1	2	3	4	5
36. Ficar facilmente incomodado ou irritado	1	2	3	4	5
37. Sentindo medo como se algo terrível pudesse acontecer.	1	2	3	4	5
**Estas perguntas são sobre como você se sente e como tem sido as coisas com você. Ao longo das últimas 4 semanas, com que frequência...**	**Nunca**	**Uma ou duas vezes**	**Algumas vezes**	**Muitas vezes**	**Frequentemente**
38. Sentir-se deprimido interferiu no que você normalmente faz?	1	2	3	4	5
39. Você se sentiu deprimido?	1	2	3	4	5
40. Você foi mal humorado ou preocupado com as coisas?	1	2	3	4	5
41. Você estava deprimido ou muito deprimido?	1	2	3	4	5
42. Você se sentiu abatido e deprimido?	1	2	3	4	5
43. Quão deprimido (no seu pior estágio) você tem sentido?	1	2	3	4	5
**Ao longo das últimas 4 semanas, quanto tempo você...**	**Nenhum tempo**	**Um pouco tempo**	**Algum tempo**	**Muito tempo**	**Todo tempo**
44. Tem dificuldade em raciocinar e resolver problemas; por exemplo, fazer planos, tomar decisões, aprender coisas novas?	1	2	3	4	5
45. Tem dificuldade em fazer atividades envolvendo concentração e pensamento?	1	2	3	4	5
46. Fica confuso e inicia várias ações ao mesmo tempo?	1	2	3	4	5
47. Esquecer, por exemplo, coisas que aconteceram recentemente, onde você colocou as coisas, compromissos?	1	2	3	4	5
48. Ter dificuldade em manter a sua atenção em qualquer atividade por muito tempo?	1	2	3	4	5
49. Reagir devagar a coisas ditas ou feitas?	1	2	3	4	5
**Quantas vezes nas últimas 4 semanas...**	**Nenhum tempo**	**Um pouco tempo**	**Algum tempo**	**Muito tempo**	**Todo tempo**
50. Você estava frustrado sobre a sua saúde?	1	2	3	4	5
51. Você se sentiu sobrecarregado pelos seus problemas de saúde?	1	2	3	4	5
52. Você se sentiu desanimado pelos seus problemas de saúde?	1	2	3	4	5
53. Você sentiu desespero sobre os seus problemas de saúde?	1	2	3	4	5
54. Você sentiu medo por causa de sua saúde?	1	2	3	4	5
55. Sua saúde foi motivo de preocupação em sua vida?	1	2	3	4	5
**As próximas perguntas são sobre como problemas de saúde podem interfeir na sua vida sexual. São perguntas pessoais, mas importantes para entender como problemas de saúde podem afetar as vidas das pessoas.**					
**Até que ponto cada um dos seguintes itens foi um problema durante as últimas 4 semanas?**	**Nenhum pouco**	**Muito pouco**	**Um pouco**	**Muito**	**Muitíssimo**
56. Falta de interesse sexual?	1	2	3	4	5
57. Incapaz de relaxar e curtir sexo?	1	2	3	4	5
58. Dificuldade em se sentir sexualmente excitado?	1	2	3	4	5

continue...

...Continuation

As últimas duas perguntas são sobre a sua vida geral.					
**Ao longo das últimas 4 semanas, até que ponto cada declaração se aplica a você?**	**Nenhum pouco**	**Muito pouco**	**Um pouco**	**Muito**	**Muitíssimo**
59. Sou capaz de curtir a vida.	1	2	3	4	5
60. Estou contente com a qualidade de minha vida no momento.	1	2	3	4	5

Você chegou ao fim desta pesquisa. Obrigado pela sua participação.
